# A Photothermally Amplified Enzyme–Nitric Oxide Co‐Regulatory System Reprograms Pathological ECM–Fibroblast Crosstalk to Alleviate Hypertrophic Scarring

**DOI:** 10.1002/advs.77376

**Published:** 2026-08-24

**Authors:** Junzhe Fu, Fan Jia, Yixian Mu, Wanzhe Liu, Dingqi Tang, Qiao Jin, Jian Ji, Youxiang Wang

**Affiliations:** ^1^ MOE Key Laboratory of Macromolecular Synthesis and Functionalization Department of Polymer Science and Engineering Zhejiang University Hangzhou People's Republic of China; ^2^ State Key Laboratory of Transvascular Implantation Devices The Second Affiliated Hospital Zhejiang University School of Medicine Hangzhou People's Republic of China; ^3^ Transvascular Implantation Devices Research Institute Hangzhou People's Republic of China

**Keywords:** ECM–fibroblast interactions, enzymatic degradation, fibrotic remodeling, hypertrophic scars, microneedles, nitric oxide

## Abstract

Hypertrophic scars (HS) are sustained by a self‐perpetuating extracellular matrix (ECM)–fibroblast feedback loop, in which activated fibroblasts drive excessive ECM deposition, while the resulting matrix stiffening in turn reinforces fibroblast activation. Although advanced fibroblast‐suppression strategies are being actively developed, this matrix‐driven profibrotic feedback remains poorly integrated into HS therapeutic strategies and insufficiently targeted. Here, we introduce an enzyme–nitric oxide (NO) co‐regulatory system for HS: enzymatic degradation of pre‐existing ECM relieves aberrant mechanical cues, while NO‐mediated modulation of fibroblast phenotypes restrains excessive collagen synthesis, thereby inducing mutually reinforcing remodeling across the ECM–fibroblast axis. Specifically, a microneedle platform is developed for localized co‐delivery of the thermosensitive protease bromelain (Bro) and NO‐donor‐functionalized polydopamine nanoparticles (PDA‐NO). Upon near‐infrared irradiation, precise photothermal stimulation further enhances Bro activity and accelerates NO release, enabling controlled amplification of the co‐regulatory effect. In vivo studies demonstrate that this system alleviates HS and restores balanced fibrotic remodeling, as evidenced by coordinated suppression of YAP signaling and the TGF‐β1/α‐SMA/Collagen I axis, supporting effective interruption of the fibrotic feedback loop. Distinct from existing fibroblast‐centric approaches, this study establishes pathological ECM–fibroblast crosstalk as a key mechanistic entry point for HS, advancing a mechanism‐driven, materials‐enabled framework for antifibrotic strategy design.

## Graphical abstract

An enzyme–nitric oxide (NO) co‐regulatory strategy reprograms pathological extracellular matrix (ECM)–fibroblast crosstalk in hypertrophic scars. Enzymatic degradation of pre‐existing ECM relieves aberrant mechanical cues, while NO‐mediated fibroblast phenotypic modulation restrains excessive collagen synthesis. Supported by photothermal amplification and localized microneedle delivery, this strategy interrupts the fibrotic feedback loop and restores balanced remodeling to alleviate hypertrophic scarring.

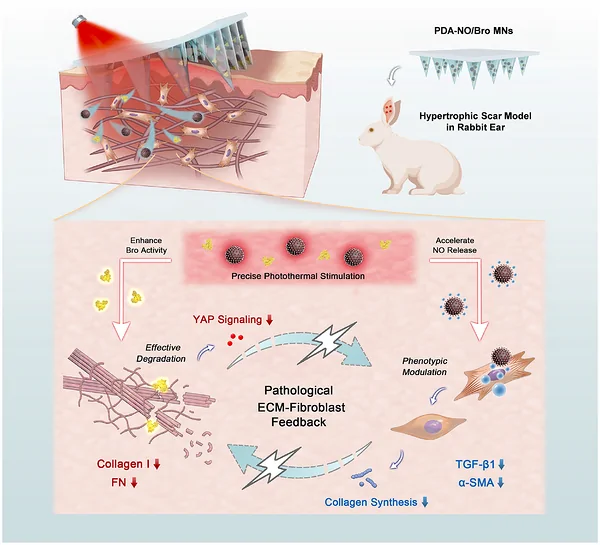

## 1 Introduction

Characterized by firm, red, and elevated lesions, hypertrophic scars (HS) arise from dysregulated wound‐healing responses following injuries to the deep dermis [1]. As a prototypical fibrotic disorder, HS have long been recognized to exhibit persistent myofibroblast activation and excessive extracellular matrix (ECM) accumulation [2, 3, 4]. Recent advances in mechanobiology have clarified the reciprocal crosstalk between these two features: myofibroblasts drive excessive ECM deposition through enhanced collagen synthesis and contractile remodeling, while the resulting mechanical cues, such as elevated tissue stiffness and strain, further sustain myofibroblast activation and reinforce their contractile phenotype [5, 6, 7, 8]. This reciprocal interaction establishes a self‐perpetuating feedback loop that fuels the progressive exacerbation of fibrosis. Identifying effective strategies to disrupt this pathological cycle has emerged as a critical yet unmet challenge in HS therapy [5].

Current treatments for HS predominantly aim to suppress aberrantly activated fibroblasts. In clinical practice, intralesional injections of 5‐fluorouracil (5‐Fu), a pyrimidine analog with antimetabolite activity, are widely employed to inhibit fibroblast proliferation and induce apoptosis [9, 10, 11, 12]. Recent studies have also explored several advanced therapeutic strategies, including photodynamic therapy and interventions that activate specific regulated cell death pathways in HS fibroblasts, such as ferroptosis and apoptosis [13, 14, 15, 16]. Although these approaches can reduce fibroblast density and attenuate ongoing collagen synthesis, they remain insufficient to interrupt the pathological ECM–fibroblast feedback. The pre‐existing stiffened matrix continues to transmit aberrant mechanotransduction signals and promote fibroblast reactivation, which may contribute substantially to the persistent and unpredictable therapeutic outcomes observed in HS. Moreover, excessive fibroblast ablation can introduce nonspecific cytotoxicity and secondary inflammation, aggravating wound‐healing responses and increasing the risk of HS recurrence. These limitations highlight the need for complementary strategy designs that place pathological ECM–fibroblast crosstalk at the center of therapeutic intervention to reprogram the self‐reinforcing fibrotic process, while ensuring safety and controllability.

In this study, we propose a dual‐action fibrotic remodeling strategy that intervenes in the ECM–fibroblast axis. One component degrades the pre‐existing ECM to relieve pathological mechanical cues, while the other noncytotoxically modulates fibroblast phenotypes to attenuate myofibroblast activation and restrain excessive collagen synthesis. By integrating these mechanistically interconnected interventions, the strategy aims to reprogram pathological ECM–fibroblast crosstalk and interrupt the self‐perpetuating fibrotic loop underlying HS progression.

**FIGURE 1:**
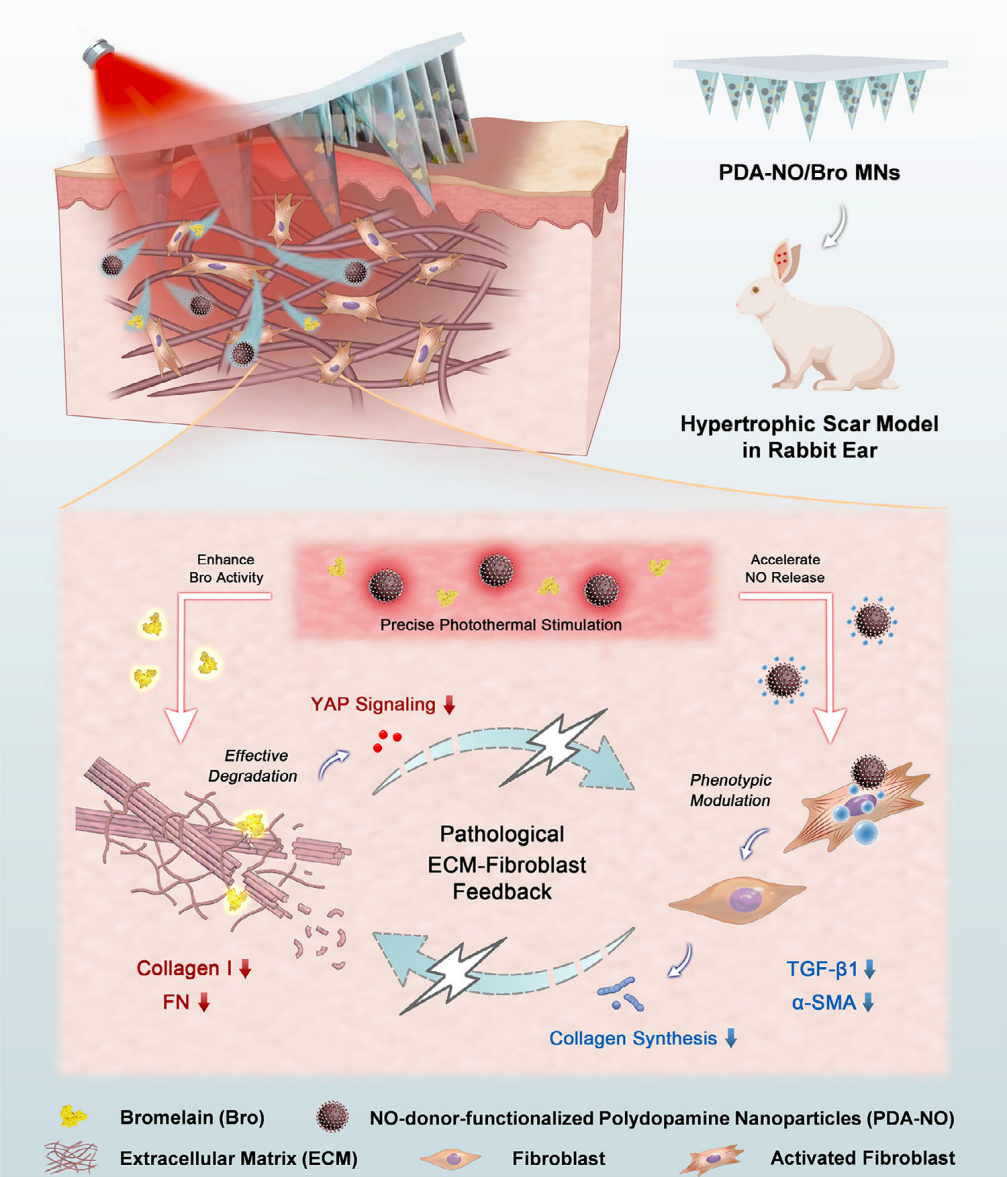
Schematic illustration of a dissolving MN platform implementing photothermally amplified enzyme–NO co‐regulation for HS therapy: localized co‐delivery of Bro and PDA‐NO enables NIR‐induced photothermal amplification of Bro‐mediated degradation of pre‐existing ECM and NO‐mediated fibroblast phenotypic modulation, thereby reprogramming pathological ECM–fibroblast crosstalk and alleviating hypertrophic scarring.

To implement this concept, we developed a microneedle (MN) platform incorporating an enzyme–nitric oxide (NO) co‐regulatory system (Figure 1). NO, a gaseous signaling molecule with well‐recognized therapeutic potential, is known to modulate the pivotal transforming growth factor‐β (TGF‐β)/Smad signaling pathway and attenuate profibrotic responses across several organ systems [17, 18, 19, 20]. Here, we applied this mechanistic insight to HS and hypothesized that NO could downregulate the activation state of myofibroblasts. Specifically, the MN platform was designed to co‐load the thermosensitive protease bromelain (Bro) and NO‐donor‐functionalized polydopamine nanoparticles (PDA‐NO). Following transdermal administration, Bro and PDA‐NO were rapidly released into the fibrotic matrix. Subsequent precisely controlled near‐infrared (NIR) irradiation activated the photothermal properties of PDA‐NO, generating mild localized heating that (i) enhanced the catalytic activity of Bro to promote ECM degradation and (ii) accelerated NO release to modulate fibroblast phenotypes, thereby strengthening their respective therapeutic effects. Together, these two actions facilitated coordinated remodeling across the ECM–fibroblast axis in a mutually reinforcing manner. In vivo experiments further demonstrated that our system restored balanced fibrotic remodeling and alleviated HS by coordinately suppressing mechanotransduction‐related Yes‐associated protein (YAP) signaling and the profibrotic TGF‐β1/α‐smooth muscle actin (α‐SMA)/type I collagen (Collagen I) axis. Notably, this work represents the first application of a therapeutic gas in HS treatment, broadening the therapeutic landscape of fibrotic intervention. More importantly, this system establishes pathological ECM–fibroblast crosstalk as a central therapeutic entry point for HS. It may serve as a mechanism‐guided, materials‐enabled platform for future antifibrotic strategy design.

## 2 Results and Discussion

### 2.1 Preparation and Characterization of PDA‐NO

**FIGURE 2:**
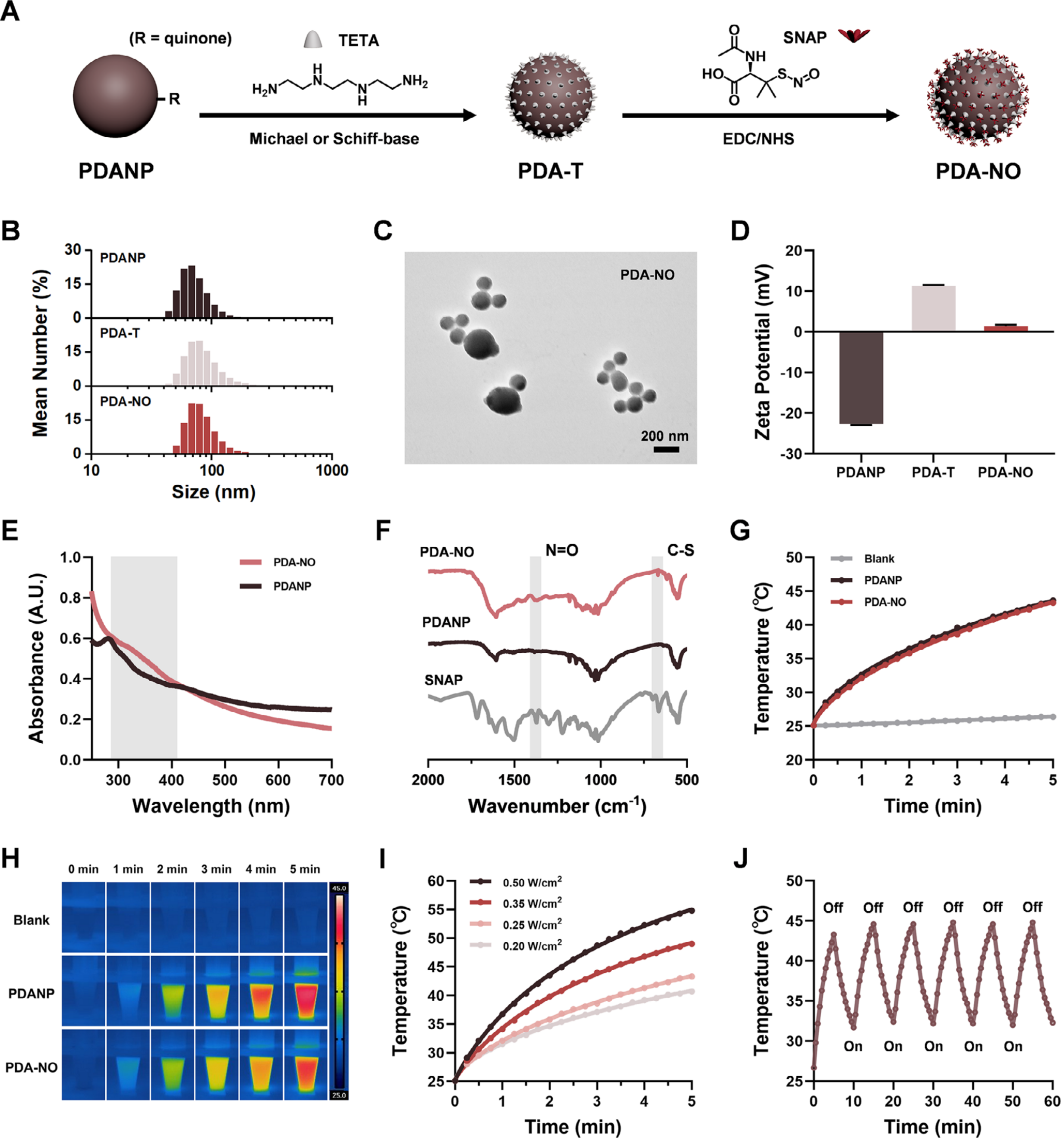
Preparation and characterization of PDA‐NO. (A) Synthesis scheme of PDA‐NO via two‐step surface functionalization. (B) DLS analysis of PDANP, PDA‐T, and PDA‐NO. (C) Representative TEM image of PDA‐NO. (D) Zeta potential measurements of PDANP, PDA‐T, and PDA‐NO. (E) UV–vis spectrum and (F) FT‐IR spectra of PDANP and PDA‐NO. (G) Photothermal curves and (H) corresponding thermal images of PDANP and PDA‐NO under 808 nm NIR laser irradiation (0.25 W cm^−2^) for 5 min. (I) Temperature increase of PDA‐NO suspension under NIR laser exposure at different irradiation power densities. (J) Photothermal stability of PDA‐NO after six consecutive heating–cooling cycles.

The PDA‐NO was synthesized through a two‐step surface functionalization process (Figure 2A and Figures S1 and S2). In the first step, pre‐synthesized polydopamine nanoparticles (PDANP) were reacted with triethylenetetramine (TETA) to enrich the particle surface with additional amine functionalities, generating PDA‐T. During this modification, the oxidized quinone moieties on the PDANP surface reacted with one of the primary amines of TETA through either Michael addition or Schiff‐base condensation. In the subsequent step, the thermally labile S‐nitrosothiol donor S‐nitroso‐N‐acetyl‐D‐penicillamine (SNAP) was covalently coupled to the remaining free amines on PDA‐T via a carbodiimide‐mediated coupling reaction, yielding the final PDA‐NO nanoconstruct. Dynamic light scattering (DLS) analysis revealed that the hydrodynamic diameters of the nanoparticles remained essentially unchanged throughout the functionalization process (Figure 2B). The final PDA‐NO displayed a uniform spherical morphology, as confirmed by transmission electron microscopy (TEM) (Figure 2C), and exhibited a number‐average hydrodynamic diameter of 85 nm with a narrow size distribution (polydispersity index, PDI = 0.164).

To verify the successful surface functionalization achieved through the two‐step synthesis, the nanoparticles were characterized using zeta potential measurements, UV–visible (UV–vis) spectroscopy, and Fourier‐transform infrared (FT‐IR) spectroscopy. Due to the introduction of additional surface amine groups, the zeta potential of PDA‐T reversed from −22.6 to +11.3 mV compared with PDANP (Figure 2D). Subsequently, the conjugation of SNAP consumed the surface amines, leading to a marked decrease in the zeta potential of PDA‐NO to nearly neutral. The UV–vis spectrum of PDA‐NO displayed a distinct absorption peak at 340 nm, corresponding to the characteristic S‐nitrosothiol group of SNAP (Figure 2E and Figure S3). The FT‐IR spectra further supported the successful surface functionalization (Figure 2F). Compared with PDANP, PDA‐NO exhibited two new characteristic absorption bands at 1374 and 667 cm^−1^, attributed to the N═O and C─S stretching vibrations of SNAP, respectively. The appearance of these new bands, which were absent in the pristine PDANP spectrum, confirmed that SNAP was successfully introduced onto the PDANP surface.

On this basis, the NO‐donating capacity of PDA‐NO was further quantified by measuring its total releasable NO content. This value was determined to be 0.929 ± 0.006 µmol mg^−1^ PDA‐NO. After storage for 1, 3, and 7 days, the total releasable NO content remained comparable to that on Day 0, with no obvious decrease, indicating that PDA‐NO exhibited good storage stability in its NO‐donating capacity (Figure S4).

After confirming the surface functionalization and NO‐donating capacity of PDA‐NO, its photothermal behavior was next investigated under 808 nm NIR laser irradiation. As expected, PDA‐NO exhibited photothermal conversion performance comparable to that of PDANP (Figure 2G,H). Moreover, PDA‐NO showed a power‐dependent temperature increase upon NIR laser exposure. When the irradiation power density was adjusted to 0.20, 0.25, 0.35, and 0.50 W cm^−2^, the temperature of the PDA‐NO suspension reached 40.7°C, 43.3°C, 49.0°C, and 54.8°C, respectively, after 5 min of irradiation, demonstrating its tunable photothermal performance for different therapeutic requirements (Figure 2I). In addition, the photothermal conversion ability of PDA‐NO remained stable over six consecutive heating–cooling cycles, indicating excellent photothermal durability (Figure 2J).

### 2.2 Determination of an Appropriate Photothermal Window for HS Intervention

**FIGURE 3:**
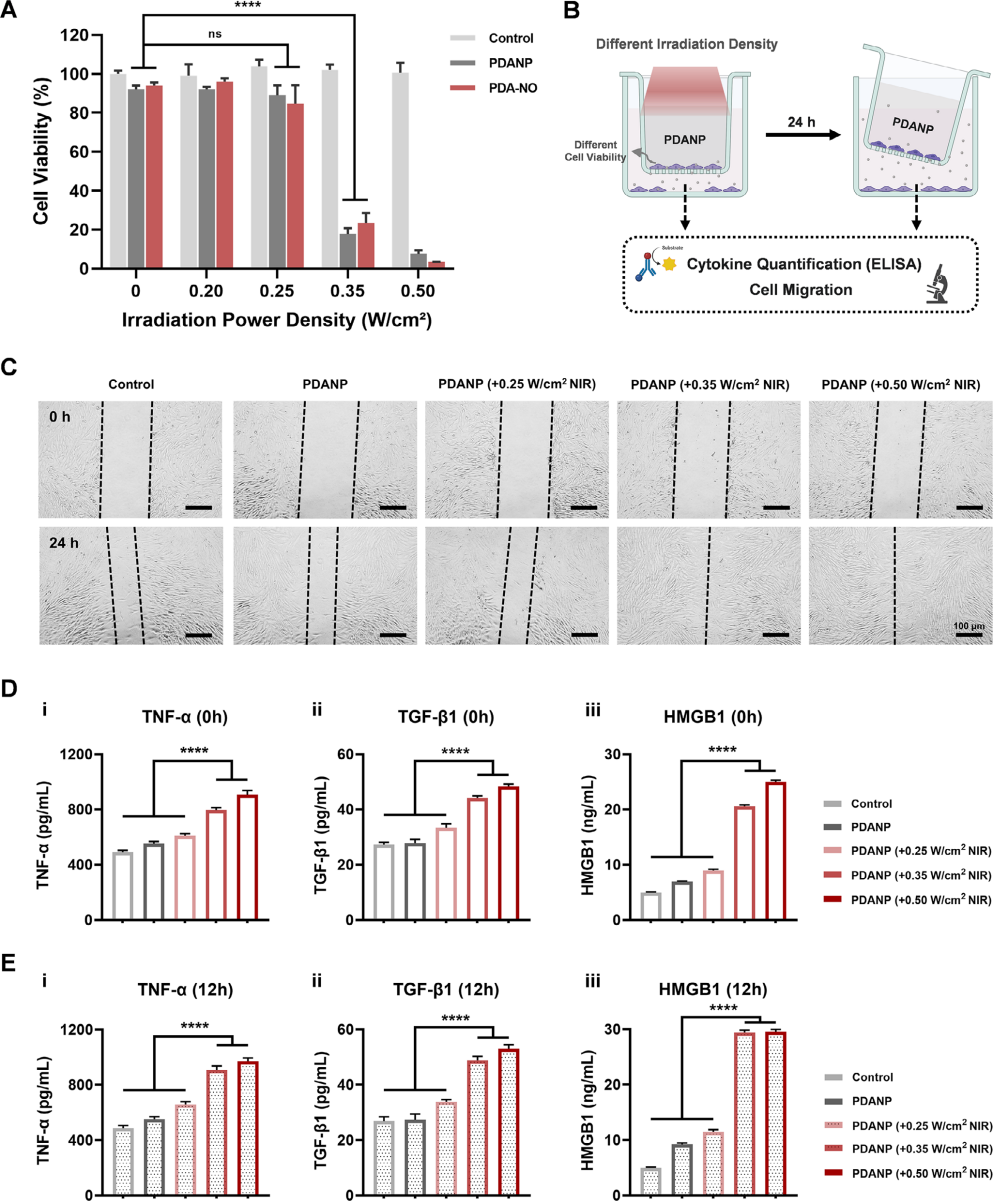
Determination of an appropriate photothermal window for HS intervention. (A) Cytotoxicity of HSFbs exposed to photothermally induced temperatures generated by different irradiation power densities (*n* = 4 biologically independent samples). (B) Schematic of the experimental setup to evaluate the effect of photothermal stimulation on HSFb response in a Transwell assay. (C) Migration of HSFbs in the lower chamber after photothermal treatment of upper‐layer cells at different power densities. (D,E) ELISA analysis of cytokine levels (TNF‐α, TGF‐β1, HMGB1) secreted by HSFbs at (D) 0 h and (E) 12 h following photothermal stimulation at different intensities (*n* = 3 biologically independent samples). ns, not significant, ^****^ *p* < 0.0001.

In our design, photothermal stimulation is intended to amplify enzyme–NO co‐regulation. Before being applied for this purpose, an appropriate photothermal window for HS intervention needed to be identified through systematic experimental evaluation. We first evaluated the photothermally induced cytotoxicity in human HS fibroblasts (HSFbs) across a range of irradiation power densities, which generated distinct temperature profiles as presented in Figure 2I. As shown in Figure 3A, when the irradiation power density was set to 0.25 W cm^−2^ (resulting in a maximum photothermal temperature below 45°C), no significant difference in cell viability was observed for either the PDANP or PDA‐NO groups. However, as the temperature further increased, cell viability markedly declined. Specifically, when the irradiation power density increased to 0.35 and 0.50 W cm^−2^, corresponding to maximum temperatures of 46°C–50°C and 51°C–55°C after 5 min of irradiation, cell viability sharply dropped to below 25% and 10%, respectively.

Subsequently, HSFbs were seeded into both the upper and lower chambers of a Transwell insert. Only the cells in the upper chamber were treated with PDANP and exposed to NIR laser irradiation, whereas the cells in the lower chamber remained untreated (Figure 3B). This compartmentalized setup was designed to model the indirect effects of localized photothermal stimulation on adjacent, nonirradiated fibroblasts. The results demonstrated that photothermal stimulation of the upper‐layer cells at 0.35 and 0.50 W cm^−2^ significantly enhanced migration of lower‐layer fibroblasts, leading to nearly complete scratch closure within 24 h (Figure 3C). In contrast, when the upper‐layer cells were treated at 0.25 W cm^−2^, fibroblast migration in the lower chamber was comparable to that of the Control group. We speculated that this effect might be mediated by paracrine signaling induced by photothermal stimulation. To verify this hypothesis, cytokine levels were quantified using ELISA at 0 and 12 h (Figure 3D,E). The expression of the pro‐inflammatory cytokine tumor necrosis factor‐α (TNF‐α), the growth factor TGF‐β1, and the damage‐associated molecular pattern (DAMP) protein high‐mobility group box 1 (HMGB1) was markedly upregulated in the PDANP (+0.35 W cm^−2^ NIR) and PDANP (+0.50 W cm^−2^ NIR) groups. Notably, this upregulation persisted from 0 to 12 h, indicating that the release of these mediators was not merely a transient event. Conversely, the expression of these factors in the PDANP (+0.25 W cm^−2^ NIR) group was only slightly higher than that in the Control group, consistent with the absence of significant enhancement in cell migration. These findings suggested that photothermal stimulation above 45°C could undesirably promote neighboring fibroblast migration by inducing a stress‐ and damage‐associated paracrine response. Excessively intense photothermal conditions appeared unfavorable for HS intervention.

To conclusively determine the optimal photothermal intensity, we employed a rabbit ear HS model and applied three temperature ranges identical to those used previously (Figures S5 and S6). During in situ photothermal treatment, maximum temperatures exceeding 45°C caused marked local secondary injury, and the irradiated regions developed deep eschars of different severities (Figure S7). After the eschars had naturally detached, histological analysis revealed that the dermal thickness of HS tissue was not reduced but instead increased, accompanied by marked aggregation and infiltration of inflammatory cells (Figure S8). Collectively, these in vivo results corroborated the in vitro findings and revealed an important feature of photothermal intervention in HS: increasing thermal intensity did not necessarily translate into a stronger fibroblast‐suppressive effect; instead, stimulation above 45°C induced a stress‐ and damage‐associated paracrine response in vitro and maladaptive tissue remodeling in vivo, suggesting a shift toward a pro‐repair and potentially profibrotic microenvironment. This finding highlighted the need to carefully control photothermal stimulation rather than simply maximizing thermal intensity. On this basis, a photothermal range of 42°C–45°C was selected as the appropriate window for subsequent experiments.

### 2.3 In Vitro Photothermal Amplification of Bro Activity and NO‐Mediated Fibroblast Phenotypic Modulation

**FIGURE 4:**
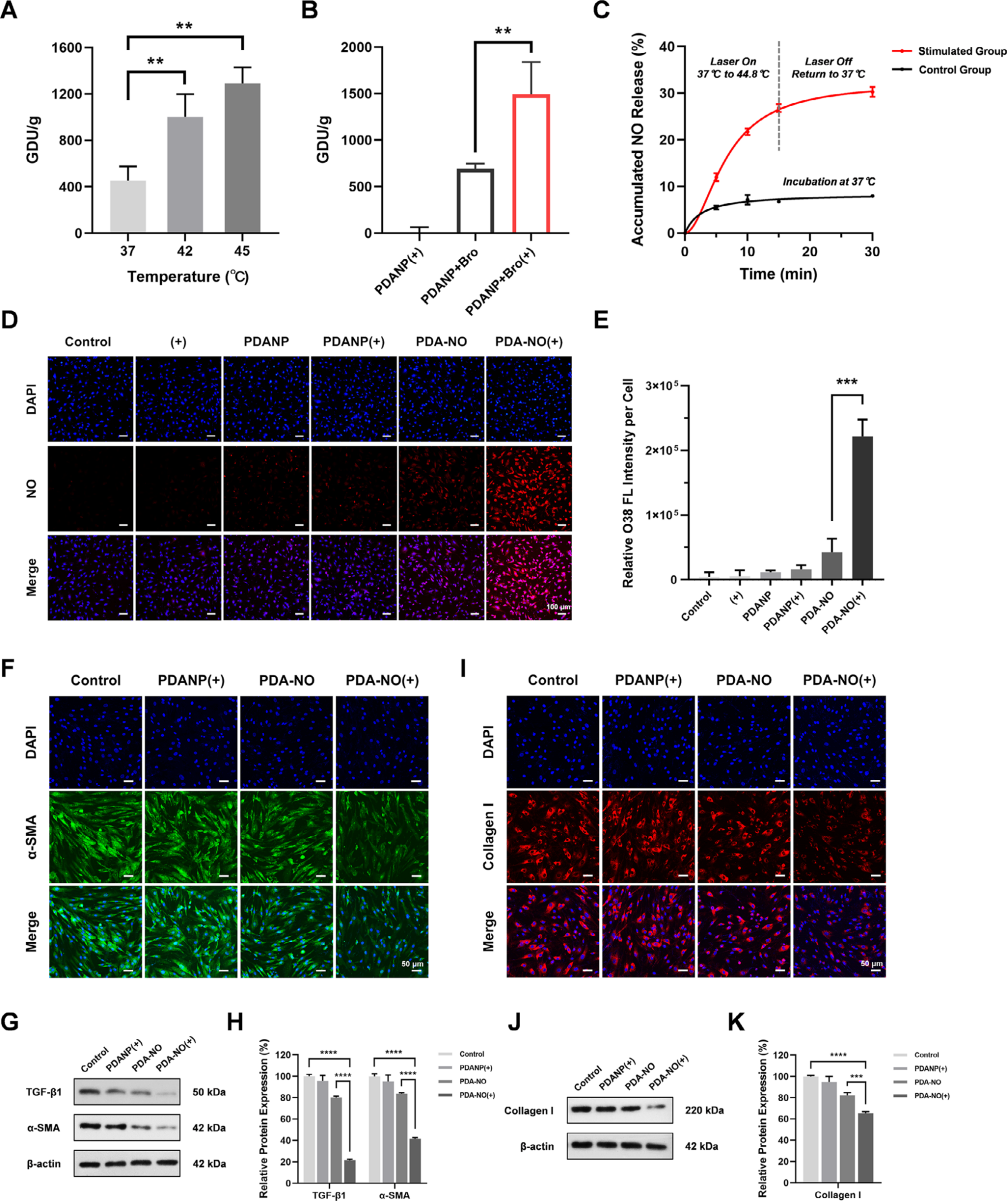
Dual photothermal amplification of Bro enzymatic activity and NO‐induced fibroblast phenotypic modulation in vitro. (A) Gelatin Digestion Unit assay of Bro at different temperatures and (B) with or without photothermal irradiation. (C) Cumulative NO release from PDA‐NO suspensions with or without photothermal stimulation. (D) Fluorescence images of intracellular NO release in HSFbs after different treatments (red fluorescence: intracellular NO visualized using probe O38; blue fluorescence: nuclei stained with DAPI), and (E) relative O38 fluorescence intensity per cell. (F) Immunofluorescence images of α‐SMA in HSFbs under different treatments. (G) Representative Western blot bands and (H) corresponding quantitative analysis of TGF‐β1 and α‐SMA expression. (I) Immunofluorescence images of Collagen I expression in HSFbs. (J) Representative Western blot bands and (K) corresponding quantitative analysis of Collagen I expression. *n* = 3 biologically independent samples. ^**^ *p* < 0.01, ^***^ *p* < 0.001, ^****^ *p* < 0.0001.

Bro, as noted previously, is a distinctive thermosensitive sulfhydryl (thiol) protease [21, 22]. Its enzymatic activity increases with temperature over a certain range and generally remains high up to approximately 50°C [23, 24, 25]. This characteristic offers the potential for remote control of its proteolytic function. To evaluate the temperature dependence of Bro, its proteolytic activity was first measured in vitro using the standard Gelatin Digestion Unit (GDU) assay [26]. As expected, Bro activity increased markedly with temperature, reaching 452.7 ± 122.3, 1003.3 ± 194.2, and 1290.3 ± 138.1 GDU/g after 20 min of incubation at 37°C, 42°C, and 45°C, respectively (Figure 4A). Subsequently, Bro was pre‐mixed with PDANP suspension and exposed to 15 min of laser irradiation, during which the temperature increased from 37°C to a maximum of 44.8°C. After the laser was turned off, the temperature naturally returned to 37°C within another 15 min. Under these conditions, the enzymatic activity of Bro was approximately 2.2‐fold higher than that of the PDANP+Bro group maintained at 37°C for 30 min (Figure 4B). These results demonstrated that the selected photothermal window could enhance the proteolytic activity of Bro, which is favorable for achieving more efficient degradation of pre‐existing ECM.

As the second component of this co‐regulation system, we further examined whether the selected photothermal window could accelerate the thermally responsive release of NO and thereby more effectively modulate fibroblast phenotypes. The cumulative release of NO from PDA‐NO suspensions was quantified using the Griess assay (Figure 4C) [27, 28]. Under the same photothermal conditions employed to assess Bro activity, the stimulated group exhibited a cumulative NO release of 26.8% after 15 min of irradiation, which increased to 30.3% at 30 min. In contrast, the Control group maintained at 37°C for 30 min released only 7.9% of the total NO. These results clearly demonstrated that photothermal stimulation effectively accelerated NO release, which was attributable to the thermolability of RSNO bonds in surface‐tethered SNAP moieties on PDA‐NO; localized heating promoted their homolytic cleavage, thereby liberating NO. Moreover, intracellular NO release was visualized using the NO‐activatable fluorescent probe O38 (Figure 4D). Negligible red fluorescence was observed in HSFbs treated with the (+), PDANP, and PDANP(+) groups, thereby ruling out NO generation by NIR irradiation or photothermal conversion alone. In comparison, the PDA‐NO group exhibited faint fluorescence signals, most likely arising from the slow thermal decomposition of RSNO moieties under 37°C. Notably, cells in the PDA‐NO(+) group displayed markedly stronger red fluorescence, approximately fivefold greater than that of the PDA‐NO group, indicating efficient cellular uptake of PDA‐NO and photothermally accelerated intracellular NO release (Figure 4E).

α‐SMA is one of the most commonly used markers for identifying fibroblast activation and phenotypic remodeling [5]. In the context of HS, its positive expression is widely recognized as an important indicator of a highly activated myofibroblast phenotype [29, 30, 31, 32]. As shown in Figure 4F, untreated HSFbs exhibited strong green fluorescence, indicating a highly activated phenotype. HSFbs treated with PDANP(+) and PDA‐NO also showed pronounced α‐SMA expression; however, fluorescence intensity was markedly reduced after PDA‐NO(+) treatment. Western blot analysis revealed a consistent trend: relative α‐SMA abundance was 83.7% of the control level in the PDA‐NO group but decreased to 41.7% with PDA‐NO(+), suggesting that photothermally accelerated NO release downregulated the activation state of HSFbs (Figure 4G,H and Figure S9). Acting upstream of α‐SMA, TGF‐β1 is one of the best‐characterized and most potent cytokines driving fibroblast activation and α‐SMA upregulation [33]. Consistent with this, PDA‐NO(+) treatment substantially reduced cellular TGF‐β1 expression, indicating that photothermally accelerated NO release produced bioactive concentrations sufficient to modulate the profibrotic phenotype of HSFbs.

In HS, Collagen I is the predominant ECM component deposited by activated myofibroblasts [2]. Having demonstrated that NO shifted HSFbs toward a more quiescent state, we next examined the expression of Collagen I. Immunofluorescence and Western blot analyses revealed that Collagen I levels were significantly reduced in the PDA‐NO(+) group (Figure 4I–K and Figure S10). Collectively, these findings indicated that photothermally accelerated NO release more effectively downregulated the activation state of HSFbs. More importantly, they revealed a previously underexplored role of NO in HSFbs in regulating TGF‐β1/α‐SMA signaling and suppressing Collagen I synthesis, underscoring its potential to drive antifibrotic remodeling along the fibroblast‐to‐ECM axis.

### 2.4 Fabrication and Characterization of the MN System

**FIGURE 5:**
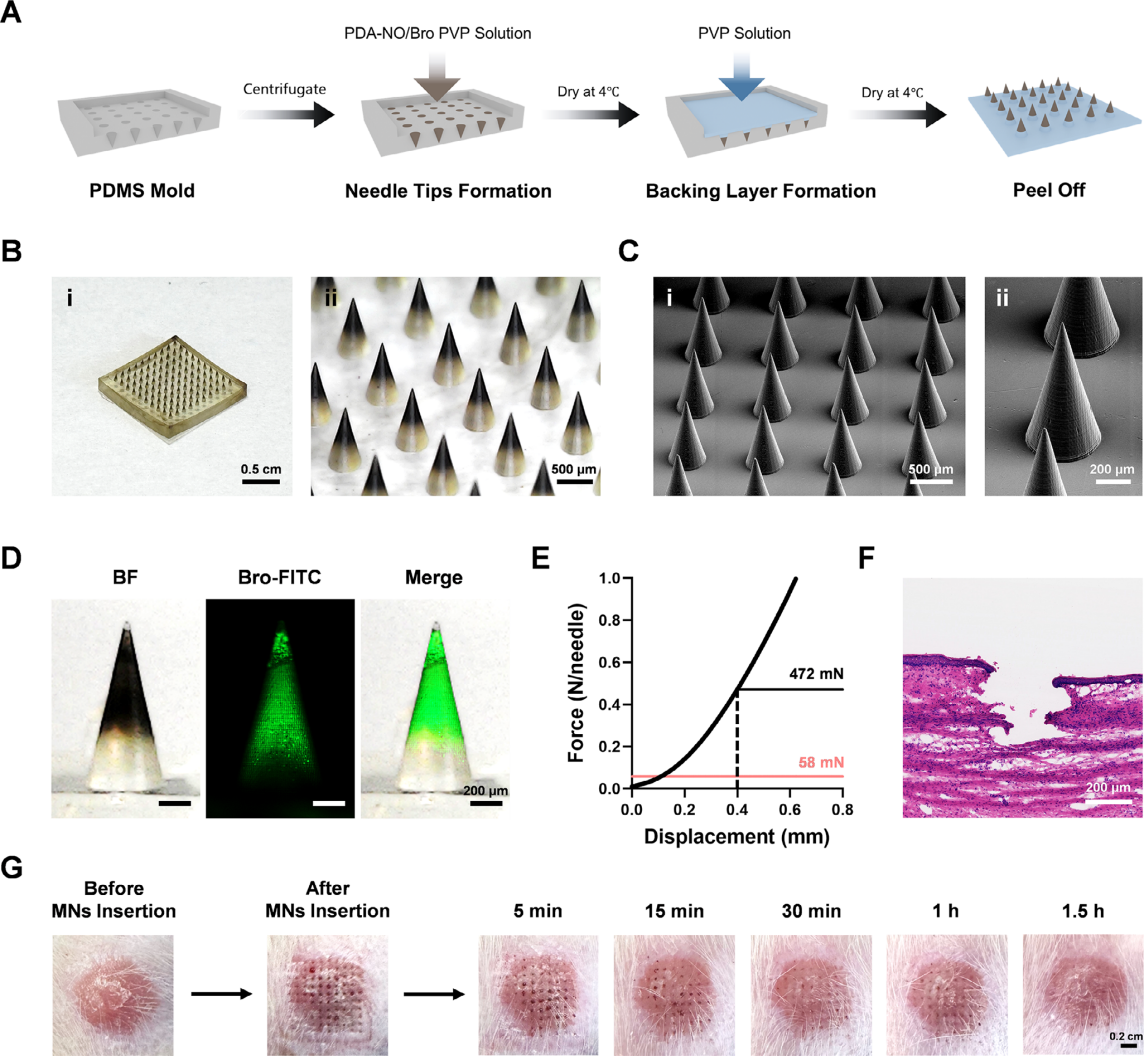
Fabrication and characterization of the MN system. (A) Workflow for the fabrication of PDA‐NO/Bro MNs. (B) Representative photograph and (C) SEM image of PDA‐NO/Bro MNs. (D) Fluorescence side‐view images showing co‐localization of FITC‐labeled Bro and PDA‐NO at the needle tip. (E) Compression force versus displacement plots of PDA‐NO/Bro MNs. (F) H&E staining of HS tissue after MN application. (G) Representative bright‐field image of HS tissue after MN insertion (top view).

Inspired by the in vitro results described above, a polymer‐based MN delivery system was developed to achieve localized co‐delivery of PDA‐NO and Bro for in vivo application. Specifically, PDA‐NO/Bro MNs was fabricated using a two‐step micromolding technique (Figure 5A). Poly(vinylpyrrolidone) (PVP) with a low molecular weight (∼58 000 Da) was selected as the primary MN matrix owing to its rapid dissolution rate and excellent biocompatibility. Representative images in Figure 5B,C showed the PDA‐NO/Bro MNs arranged in a 10 × 10 array, featuring conical needles with a height of 1000 µm and a base diameter of 500 µm. The full‐array bright‐field stereomicroscopy image also showed consistent tip‐localized dark PDA‐NO‐enriched regions across the array, with no obvious needle‐to‐needle heterogeneity (Figure S11). To further investigate the co‐localization of Bro with PDA‐NO, Bro was conjugated with fluorescein isothiocyanate (FITC) for fluorescence microscopy observation (Figure S12). As shown in Figure 5D, the green fluorescence signal from FITC‐Bro was spatially consistent with the dark PDA‐NO regions visible under bright‐field imaging, indicating uniform co‐localization and enrichment of both agents at the needle tips.

Since adequate mechanical strength is critical for skin insertion, a compression test was conducted to assess the MNs’ mechanical performance. The mechanical strength of the PDA‐NO/Bro MNs was determined to be 472 mN per needle at a displacement of 400 µm, which greatly exceeded the minimum requirement for skin penetration (58 mN) (Figure 5E) [34]. This mechanical robustness was further validated by a penetration test using HS tissue. Histological analysis confirmed that the MN successfully penetrated the stratum corneum with an insertion depth of approximately 350 µm (Figure 5F). Furthermore, as shown in the bright‐field image of HS tissue in Figure 5G, a clear 10 × 10 array of tip imprints was observed from the top view after MNs administration, indicating precise and uniform insertion. Additionally, the microspots created on the skin surface gradually disappeared within 1.5 h without causing any secondary injuries.

### 2.5 In Vivo Therapeutic Efficacy of the MN System on HS

**FIGURE 6:**
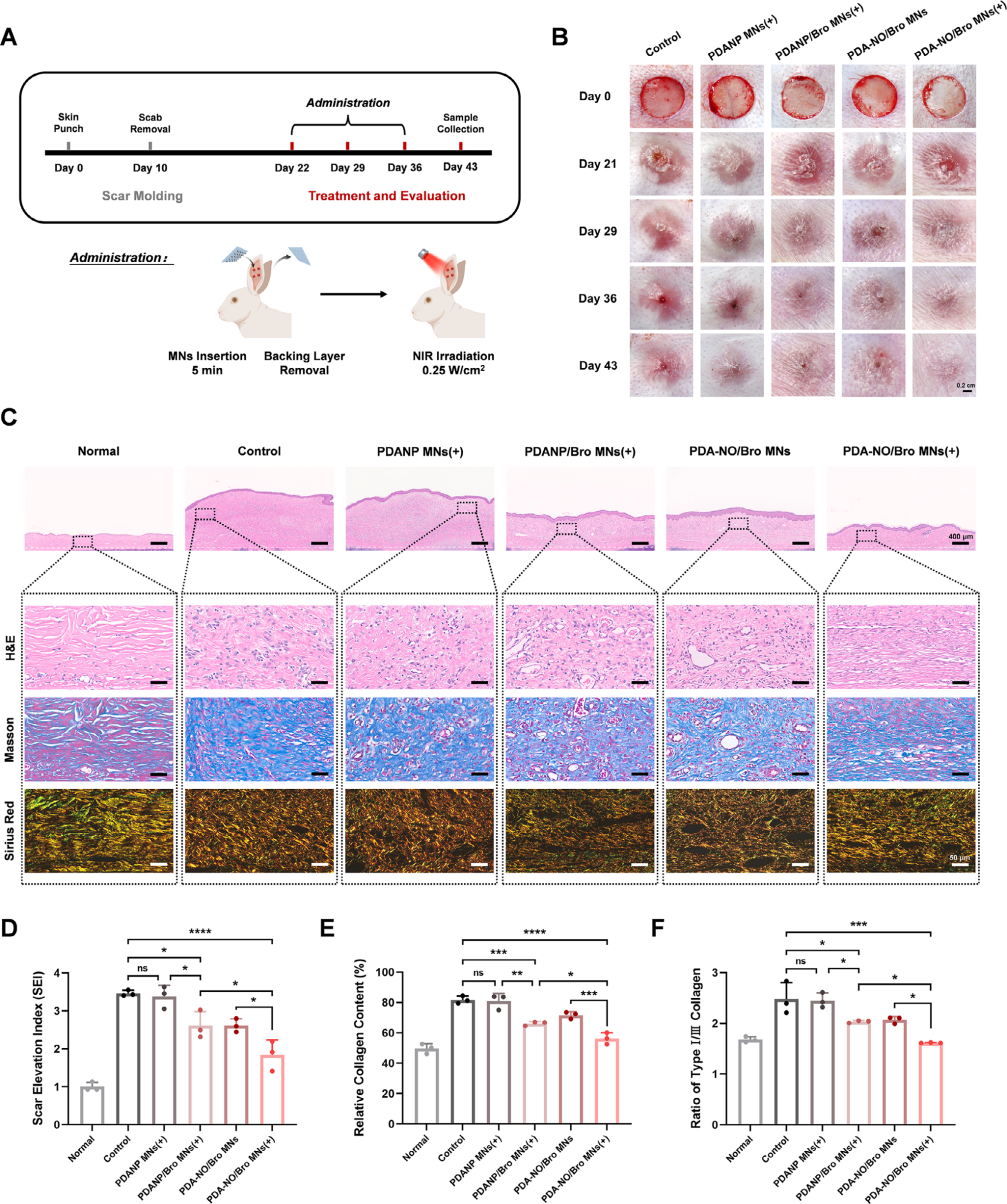
In vivo anti‐scarring evaluation on the rabbit ear HS model. (A) Timeline and administration scheme of the experimental process. (B) Photographs of the wound on Day 0 and HS lesions at different time points (Day 21, 29, 36, and 43). (C) Representative images of H&E staining, Masson's trichrome staining, and Sirius Red staining of HS on Day 43. (D–F) Quantitative analysis of (D) SEI, (E) relative collagen content, and (F) type I/III collagen ratio based on staining images (*n* = 3 biologically independent animals per group). ns, not significant, ^*^ *p* < 0.05, ^**^ *p* < 0.01, ^***^ *p* < 0.001, ^****^ *p* < 0.0001.

After successfully fabricating the MN system, in vivo experiments were conducted to evaluate its anti‐scar efficacy (Figure 6A). As in the previously described photothermal intensity evaluation, a rabbit ear HS model was established through full‐thickness skin excision followed by delayed epithelialization, which was completed on Day 21. The rabbits were then randomly divided into five groups: (1) Control group, receiving no treatment; (2) PDANP MNs(+) group, treated with photothermal stimulation alone; (3) PDANP/Bro MNs(+) group, treated with Bro under photothermal stimulation to enhance Bro‐mediated ECM degradation; (4) PDA‐NO/Bro MNs group, treated with PDA‐NO and Bro without NIR irradiation; and (5) PDA‐NO/Bro MNs(+) group, treated with PDA‐NO and Bro under photothermal stimulation to amplify the enzyme–NO co‐regulatory effect. Given the rapid dissolution rate of the MN matrix, the backing layer could be removed 5 min after insertion, allowing the needle tips to fully dissolve within the HS tissue (Figures S13 and S14). In accordance with the previously optimized photothermal window, when irradiated at 0.25 W cm^−2^, the maximum temperature in groups (2), (3), and (5) was maintained at 42°C–45°C. Treatments were administered once per week starting on Day 22, and samples were collected on Day 43 for subsequent analysis. Body weights were also monitored throughout the study and showed only minor transient fluctuations with an overall stable‐to‐increasing trend, indicating that the treatment regimen was generally well tolerated (Figure S15).

As shown in Figure 6B, compared with the Control group, the PDANP MNs(+) group exhibited negligible therapeutic efficacy after three administrations, with the HS remaining raised, firm, and reddish. Encouragingly, the lesions in the other three treatment groups displayed a visible trend toward morphological normalization. Among them, the PDANP/Bro MNs(+) group presented flattened and softened lesions, suggesting that Bro contributed to the remodeling of excessively deposited ECM under photothermal stimulation. The therapeutic effect was even more pronounced in the PDA‐NO/Bro MNs(+) group, indicating that NO‐mediated fibroblast phenotypic modulation cooperated with enzymatic ECM degradation to improve HS remodeling. Moreover, compared with the PDA‐NO/Bro MNs group, the PDA‐NO/Bro MNs(+) group exhibited lesion colors that more closely resembled normal skin and showed less distinct lesion boundaries. This comparison supported the role of photothermal amplification in strengthening the overall in vivo effects of the PDA‐NO/Bro MN system. Hematoxylin and eosin (H&E), Masson's trichrome, and Sirius Red staining highlighted the histological changes in HS lesions following different treatments (Figure 6C). Consistent with the observed therapeutic efficacy, PDANP/Bro MNs(+) treatment substantially reduced collagen deposition relative to PDANP MNs(+), while the ECM architecture was further normalized in the PDA‐NO/Bro MNs(+) group, where collagen fibers were arranged more loosely and in a more organized manner. Quantitative analysis further revealed that the PDA‐NO/Bro MNs(+) group exhibited the lowest scar elevation index (SEI), the lowest relative collagen content, and the smallest type I/III collagen ratio among all groups (Figure 6D–F).

**FIGURE 7:**
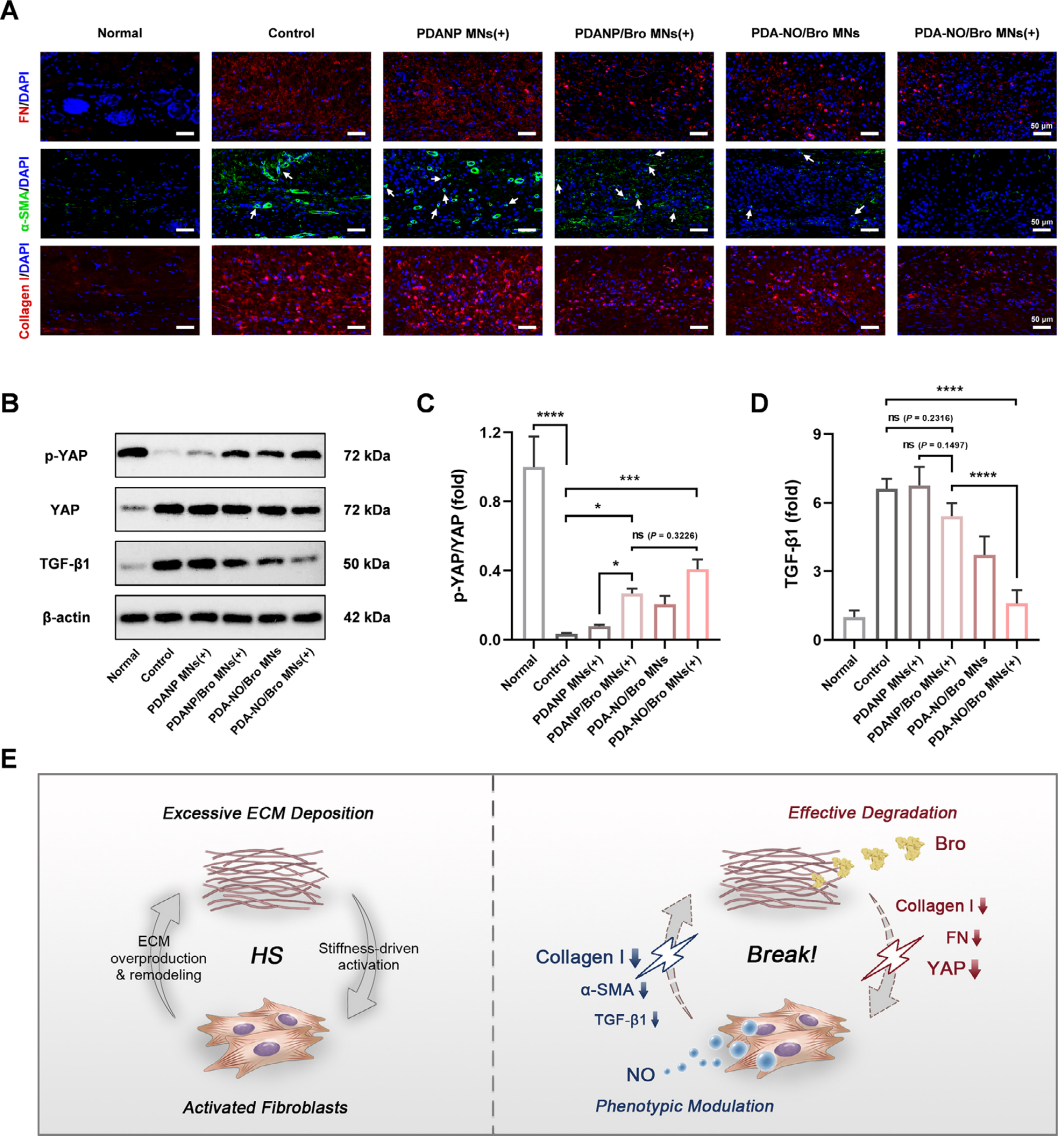
In vivo analysis of fibrotic remodeling, YAP signaling, and profibrotic pathways in rabbit ear HS. (A) Representative immunofluorescence staining of FN, α‐SMA, and Collagen I of HS from each group on Day 43. (B) Representative Western blot bands for p‐YAP, YAP, and TGF‐β1. (C,D) Quantification of (C) p‐YAP/YAP ratio and (D) TGF‐β1 expression (*n* = 3 biologically independent animals per group). (E) Schematic illustration of the enzyme‐NO‐coordinated strategy interrupting the profibrotic feedback loop. ns, not significant, ^*^ *p* < 0.05, ^***^ *p* < 0.001, ^****^ *p* < 0.0001.

Subsequently, Western blotting and immunofluorescence staining were performed to elucidate how Bro and NO mediated their regulatory effects in vivo. Fibronectin (FN) is a prominent ECM glycoprotein that mediates cell–ECM adhesion and mechanotransduction and constitutes, alongside Collagen I, a characteristic component of the excessively deposited ECM in HS [35]. Consistent with the histological findings described above, treatment with PDANP/Bro MNs(+) resulted in a pronounced reduction in FN and Collagen I expression, providing additional evidence of the degradation and remodeling of pathological ECM components in vivo (Figure 7A). To assess whether this ECM degradation relieved aberrant mechanical cues in the HS microenvironment, we focused on YAP, a key mechanotransduction effector that responds to ECM stiffness and regulates fibroblast activation and profibrotic transcriptional programs [36]. Phosphorylated YAP (p‐YAP) represents the inactive form retained in the cytoplasm; accordingly, the p‐YAP/YAP ratio serves as an inverse indicator of YAP activation. As shown in Figure 7B,C and Figure S16, compared with normal tissue, the p‐YAP/YAP ratio in HS tissue (Control group) was significantly decreased, whereas treatment with PDANP/Bro MNs(+) led to a re‐elevation of this ratio. Together with the reduction of FN and Collagen I, this result suggested that Bro‐mediated ECM remodeling contributed to the attenuation of pathological mechanotransduction along the ECM‐to‐fibroblast axis. Moreover, Figure 7D showed that TGF‐β1 expression was further decreased in the PDA‐NO/Bro MNs(+) group compared with the PDANP/Bro MNs(+) group. Consistently, immunofluorescence staining revealed markedly lower α‐SMA and Collagen I signals in the PDA‐NO/Bro MNs(+) group, in line with the cellular findings described above. These observations indicated that NO attenuated the profibrotic TGF‐β1/α‐SMA/Collagen I signaling axis on the fibroblast‐to‐ECM regulatory side.

Interestingly, the regulatory effects of Bro and NO appeared to be further interconnected. In the PDANP/Bro MNs(+) group, Bro‐mediated ECM remodeling was accompanied not only by the re‐elevation of the p‐YAP/YAP ratio, but also by modest decreases in TGF‐β1 and α‐SMA expression. This trend implied that ECM‐side remodeling had begun to dampen fibroblast‐associated profibrotic signaling. With the additional introduction of NO, TGF‐β1, α‐SMA, and Collagen I levels were more strongly suppressed. This was accompanied by a slight increase in the p‐YAP/YAP ratio relative to PDANP/Bro MNs(+), indicating that NO‐mediated suppression of fibroblast activation and collagen production could feed back onto ECM‐associated mechanotransduction. This further suggested that the two interventions acted in a mutually reinforcing manner to promote coordinated remodeling. These observations provided additional, albeit indirect, evidence for the presence of a self‐reinforcing ECM–fibroblast feedback loop in HS tissue. By targeting this loop, our strategy attenuated YAP signaling and the TGF‐β1/α‐SMA/Collagen I axis through interdependent modulation, ultimately supporting pathological crosstalk reprogramming and HS alleviation (Figure 7E).

## 3 Conclusion

In this study, we present a photothermally amplified enzyme–nitric oxide (NO) co‐regulatory system that reprograms pathological ECM–fibroblast crosstalk to alleviate HS. We engineer this system by co‐loading the thermosensitive protease Bro and PDA‐NO nanoparticles into a dissolving MN patch. Specifically, polydopamine nanoparticles are functionalized with the NO donor SNAP through a two‐step surface modification process, yielding PDA‐NO with photothermal responsiveness and thermally accelerated NO release. A photothermal window of 42°C–45°C is essential to enhance Bro activity and NO release while avoiding excessive heating that can trigger undesired profibrotic responses. Supported by both in vitro and in vivo results, this photothermal amplification simultaneously enhances Bro‐mediated enzymatic degradation of the pathological ECM and NO‐mediated modulation of fibroblast phenotypes, thereby enabling interdependent regulation of the ECM–fibroblast circuit. Mechanistically, this dual intervention interrupts the vicious cycle of mechanical and biochemical cues, as supported by the coupled attenuation of the mechanosensitive YAP signaling and the profibrotic TGF‐β1/α‐SMA/Collagen I pathway. To our knowledge, this work represents the first demonstration of a framework for targeting pathological ECM–fibroblast crosstalk as an integrated therapeutic axis in HS, providing a mechanistic basis for antifibrotic strategy design. For broader practical implementation, further optimization may focus on formulation‐ and administration‐related parameters, including long‐term Bro activity retention in the MN system, batch‐to‐batch reproducibility and longer‐term NO release stability of PDA‐NO, and the safety and practicality of extended or larger‐area repeated administration. Addressing these parameters may further improve the robustness and reproducibility of this ECM–fibroblast crosstalk‐targeted strategy, thereby supporting its development as a practical approach for fibrotic feedback modulation.

## 4 Experimental Section

### 4.1 Materials

Dopamine hydrochloride, TETA, N‐hydroxysuccinimide (NHS), PVP (M_w_ = 58 kDa), and gelatin (Bloom strength ∼250 g) were purchased from Aladdin. 1‐Ethyl‐3‐(3‐dimethylaminopropyl)carbodiimide hydrochloride (EDC·HCl) was obtained from Macklin. N‐acetyl‐D‐penicillamine and bromelain were purchased from Sigma–Aldrich. High‐temperature‐resistance photosensitive resin master molds for MN fabrication (needle height: 1000 µm, base width: 500 µm, needle spacing: 1 mm, 10 × 10 array) were supplied by BMF Precision Tech. Polydimethylsiloxane (PDMS; SYLGARD 184 silicone elastomer kit) was purchased from DOWSIL. Optimum cutting temperature (OCT) compound was purchased from Sakura Finetek. 2301 culture medium was purchased from Sciencell. The nitric oxide assay kit and cell counting kit‐8 (CCK‐8) were purchased from Beyotime. BBoxiProbe O38 solution (a nitric oxide indicator) was purchased from BestBio. The α‐SMA monoclonal antibody (RRID:AB_262054) and Collagen I polyclonal antibody (RRID:AB_3750986) used for in vitro immunofluorescence staining of HSFbs were obtained from Abcam and Arigo Biolaboratories, respectively. The FN polyclonal antibody (RRID:AB_2105691), α‐SMA polyclonal antibody (RRID:AB_2223009), and Collagen I monoclonal antibody (RRID:AB_2882107) used for immunofluorescence staining of HS tissues were purchased from Proteintech. All other reagents and solvents were of analytical grade and used as received without further purification.

### 4.2 Cells and Animals

Primary HSFbs were purchased from Hangzhou Meisen Cell Biotechnology Co., Ltd. (Cat# CTCC‐203‐HUM), and passages 3 to 6 were used in the experiments. The cells were cultured in 2301 culture medium in an incubator maintained at 37°C with 5% carbon dioxide. Female New Zealand White rabbits (*Oryctolagus cuniculus*, RRID:NCBITaxon_9986) weighing 2.0–2.5 kg were obtained from Hangzhou Yuhang Kelian Rabbit Industry Professional Cooperative. All animal experiments were conducted in strict accordance with the “Principles of Laboratory Animal Care” (NIH publication no. 86‐23, revised 1985), and all protocols were approved by the Institutional Animal Care and Use Committee, Zhejiang Center of Laboratory Animals (Approval number: ZJCLA‐IACUC‐20011138).

### 4.3 Synthesis of SNAP

N‐acetyl‐D‐penicillamine (1 g) was dissolved in methanol (8 mL). Under vigorous stirring, tert‐butyl nitrite (2 mL) was added dropwise, and the reaction mixture was allowed to proceed in the open flask for 1 min. The flask was then sealed and stirred overnight at 20°C in the dark. The solvent and by‐products were removed by rotary evaporation, and the residue was dried under vacuum to afford the final product (SNAP). The structure of the synthesized SNAP was confirmed by ^1^H NMR spectroscopy (AVANCE NEO, Bruker) (Figure S17).

### 4.4 Synthesis of PDANP

PDANP was prepared via the chemical oxidation of dopamine hydrochloride according to previously reported protocols [37]. Briefly, dopamine hydrochloride (90 mg) was dissolved in deionized water (45 mL). Under continuous stirring at 25°C, sodium hydroxide solution (1 N, 475 µL) was added dropwise. The solution color rapidly changed from colorless to light brown and gradually deepened to dark brown after 4 h of reaction. The obtained PDANP were purified using a 3000 Da ultrafiltration centrifuge tube and stored at 4°C until use.

### 4.5 Preparation and Characterization of PDA‐NO

PDANP were first functionalized with TETA to introduce additional surface amine groups. PDANP were dispersed in deionized water at 1 mg mL^−1^, and the pH was adjusted to 8.5. An excess of TETA was then added at a mass ratio of 1:9.55 (PDANP: TETA). The suspension was stirred overnight at room temperature in the dark. The resulting PDA‐T were collected by centrifugation and washed repeatedly with methanol to remove unreacted TETA. After the final wash, PDA‐T were resuspended in methanol and stored at 4°C.

In the second step, SNAP was covalently coupled to the surface amines of PDA‐T via a carbodiimide‐mediated condensation. SNAP was dissolved in methanol at 5 mg mL^−1^, followed by sequential addition of EDC·HCl and NHS at a molar ratio of 1:1.2:1.5 (SNAP: EDC: NHS). The mixture was stirred for 3 h at room temperature in the dark to generate the activated ester. PDA‐T was then added to yield a nanoparticle concentration of 0.5 mg mL^−1^ (corresponding to a SNAP: PDA‐T mass ratio of 10:1). The reaction proceeded overnight at 4°C in the dark. The product (PDA‐NO) was collected by centrifugation, thoroughly washed with methanol to remove unbound reagents, and stored at −20°C. The number‐average hydrodynamic diameters for PDANP, PDA‐T, and PDA‐NO were determined from number‐weighted size distributions obtained by DLS on a size‐zeta analyzer (ZCEC; Malvern Instruments). The ζ‐potential was measured by electrophoretic light scattering (ELS) using the Smoluchowski model on the same instrument. The microstructure and primary particle dimensions were examined by TEM (HT7800, Hitachi). Successful surface functionalization was verified by FTIR (Nicolet 6700, Thermo Fisher Scientific) and UV–vis (UV‐8000, Metash).

### 4.6 Photothermal Performance Measurement

PDA‐NO or PDANP were dispersed in aqueous solution at a consistent concentration and continuously exposed to an 808 nm laser at varying power levels. The temperature change was recorded using an infrared thermal imaging camera (FLIR E60, FLIR Systems Inc.).

### 4.7 In Vitro Cytotoxicity Assay

The CCK‐8 assay was performed to evaluate the effects of photothermally induced temperature changes on HSFbs. Specifically, HSFbs were seeded in 96‐well plates at a density of 5000 cells per well. After 18 h of incubation, the culture medium was replaced with fresh medium containing PDANP or PDA‐NO at a final concentration of 50 µg mL^−1^ and incubated for an additional 3 h to facilitate particle internalization. The cells were subsequently exposed to an 808 nm laser at different power densities (0.20, 0.25, 0.35, and 0.50 W cm^−2^) through the medium. The corresponding maximum photothermal temperatures reached 38°C–41°C, 42°C–45°C, 46°C–50°C, and 51°C–55°C, as monitored by an infrared thermal imaging camera. After a further 3 h incubation at 37°C, cell viability was determined using the CCK‐8 assay. Quantitative results were expressed as mean values (*n* = 4 biologically independent samples).

### 4.8 In Vitro Transwell Assay

For ELISA analysis, HSFbs were seeded in the upper chamber of a Transwell insert and incubated for 18 h. After incubation, the lower chamber was filled with fresh culture medium, while the cells in the upper chamber were treated with PDANP and exposed to NIR laser irradiation at intensities consistent with those used in the cytotoxicity assays. Upon completion of irradiation, the medium from the lower chamber was collected immediately and again after 12 h. The concentrations of TNF‐α, TGF‐β1, and HMGB1 in the collected samples were quantified using ELISA. For the migration assay, HSFbs were seeded in both the upper and lower chambers and incubated for 18 h. The lower‐chamber cells were scratched vertically with a 200‐µL pipette tip to create a linear wound, followed by replacement with fresh medium. The upper‐chamber cells were then treated with PDANP and subjected to NIR laser irradiation under the same conditions as described above. After 24 h, cell migration in the lower chamber was examined using an inverted microscope in bright‐field mode (ECLIPSE Ts2, Nikon).

### 4.9 Gelatin Digestion Unit

The enzymatic activity of Bro was assessed using the GDU assay, as previously described [23, 26]. For temperature‐dependent measurements, Bro was dissolved in deionized water to a concentration of 1 mg mL^−1^, and a sterile gelatin stock solution (5% w/v, 0.05 g mL^−1^) was prepared in PBS. 25 mL aliquots of the gelatin solution were equilibrated to the target temperature. For test samples, 1 mL of the Bro solution was added to the gelatin solution, mixed thoroughly, and incubated for 20 min at the target temperature. The reaction was then quenched by adding 0.10 mL of 3% H_2_O_2_ and holding the mixture for 5 min. For blank controls, 0.10 mL of 3% H_2_O_2_ was initially added and incubated for 20 min to inactivate Bro. Subsequently, 1 mL of Bro solution was added, and the mixture was held for 5 min to ensure consistent composition and timing with the test samples. The pH was then adjusted to 6.0 using HCl. Under gentle stirring, 10 mL of 37% formaldehyde was added dropwise to derivatize the liberated amino groups into Schiff bases. Finally, the mixtures were titrated to pH 9.0 with 0.1 N NaOH, and the titrant volumes (*V*_test_ and *V*_blank_) for the test and blank samples were recorded. To further examine the photothermal effect on Bro activity, PDANP suspension was added to the gelatin solution at the beginning of the procedure. The samples were exposed to 15 min of laser irradiation, during which the temperature increased from 37°C to a maximum of 44.8°C. After the laser was turned off, the temperature naturally returned to 37°C within 15 min. All other steps were performed as described previously. Enzymatic activity was calculated as follows:

(1) $$GDU=\frac{\left(V_{test}-V_{blank}\right)\times 14\times N}{m_{Bro}}$$

where *V*_test_ and *V*_blank_ represent the volume of titrant used in the test and blank samples (in mL), respectively; 14 is the constant, representing mg of nitrogen per millimole of nitrogen; *N* represents the normality of NaOH; and *m*_Bro_ is the mass of Bro used. In this experiment, *N* = 0.1 mol L^−1^, *m*_Bro_ = 0.001 g, as detailed in the experimental procedure.

### 4.10 In Vitro and Intracellular NO Release

The release of NO from PDA‐NO suspensions was quantified by measuring nitrite concentrations using the Griess reaction. The PDA‐NO suspension in the Control group was incubated at 37°C for 30 min, whereas the experimental group was exposed to 15 min of laser irradiation, during which the temperature increased from 37°C to a maximum of 44.8°C. After the laser was turned off, the temperature naturally returned to 37°C within another 15 min. Samples were collected at predetermined time intervals. Subsequently, 50 µL of the diluted sample solution, 50 µL of Griess reagent I, and 50 µL of Griess reagent II were sequentially added to a 96‐well plate. The absorbance at 540 nm was measured using a microplate reader (UV‐2550, Shimadzu), and NO release was quantified according to the calibration curve (Figure S18).

To further investigate intracellular NO release, the NO probe O38 was employed to detect NO within HSFbs. Briefly, HSFbs were seeded in 24‐well plates at a density of 4 × 10^4^ cells per well. After incubating for 18 h, the culture medium was removed, and the diluted O38 working solution was added to the wells. The plates were then incubated in the dark for 30 min. The solution in each well was replaced with the corresponding medium containing PDANP or PDA‐NO, and the plates were incubated in the dark for an additional 3 h to allow particle uptake. Subsequently, the samples were irradiated with a power of 0.25 W cm^−2^ to trigger the photothermal effect and release NO from PDA‐NO. After washing with PBS, the cells were fixed with 4% paraformaldehyde, and the cell nuclei were stained with DAPI. Imaging was performed using an inverted fluorescence microscope (ECLIPSE Ti2‐E, Nikon).

### 4.11 In Vitro Immunofluorescence Staining of HSFbs

For immunofluorescence staining, HSFbs were seeded on Φ14 mm glass coverslips in 24‐well plates at a density of 5 × 10^4^ cells per well. After incubation for 18 h, the culture medium was replaced with fresh medium containing PDANP or PDA‐NO and incubated for an additional 3 h to promote particle internalization. Subsequently, the samples were irradiated at a power density of 0.25 W cm^−2^ and then further incubated for 12 h. The cells were then fixed with 4% paraformaldehyde and permeabilized with 0.1% Triton X‐100 to increase cell membrane permeability. Afterward, the samples were blocked with 0.1% BSA in TBS buffer for 1 h at room temperature to prevent nonspecific binding. Following blocking, the samples were incubated with the diluted primary antibody solution overnight at 4°C, followed by incubation with a diluted secondary antibody for 1 h at room temperature in the dark. For nuclear staining, the samples were treated with 1 µg mL^−1^ DAPI solution for 30 min at room temperature in the dark. Finally, an anti‐fade mounting medium was added, and fluorescence images were acquired using an inverted fluorescence microscope.

### 4.12 Preparation of PDA‐NO/Bro MNs

The PDMS inverse molds were prepared by casting a PDMS mixture (base‐to‐curing agent ratio of 10:1, w/w) onto a resin master mold and curing it at 80°C. The PDA‐NO/Bro MNs was fabricated using a two‐step micromolding technique. In the first step, a 50% (w/v) PVP solution containing PDA‐NO (3 mg mL^−1^) and Bro (40 mg mL^−1^) was introduced into the PDMS mold by centrifugation (5000 rpm, 5 min). After removing the residual solution, the needle tips were dried in a desiccator for 1 h. In the second step, a pure 100% (w/v) PVP solution was cast, centrifuged (3000 rpm, 3 min), and dried for 24 h to form the backing layer of the MN patch. Notably, the entire centrifugation and drying processes were conducted at 4°C under light‐protected conditions to help preserve Bro activity and minimize premature NO release. The resulting PDA‐NO/Bro MNs were then peeled off from the mold, sealed in light‐protected self‐sealing bags containing desiccant, and also stored at 4°C before use. The morphology of the MN patch was examined using a digital microscope, a stereomicroscope (AZ100, Nikon), and a scanning electron microscope (SEM, SU8600, Hitachi).

### 4.13 Visualization of Payload Distribution

To investigate the distribution of PDA‐NO and Bro in MNs, Bro was conjugated with FITC for fluorescence microscopy observation. This conjugation is based on a nucleophilic addition reaction between the isothiocyanate group (‐N═C═S) of FITC and the amino group (‐NH_2_) of the protein, forming a stable thiourea bond. Specifically, FITC and Bro were dissolved in a PBS buffer (pH 8.0–8.2) at a molar ratio of 10:1 and reacted in the dark at room temperature for 4 h. Afterward, gradient dialysis was used to remove any unreacted free FITC. To verify successful labeling, UV–vis spectroscopy was performed across the 200–600 nm range, measuring absorbance at 275 nm (protein peak) and 493 nm (FITC peak). The labeling efficiency (degree of labeling, DOL) was then calculated based on well‐established principles from spectroscopy and protein quantification. Finally, PDA‐NO/Bro‐FITC MNs was prepared and observed using a digital microscope and a laser scanning confocal microscope (LSM 900, Zeiss).

### 4.14 Mechanical Strength and Skin Penetration Test

The mechanical strength of the MN patch was evaluated using an electromechanical universal testing system (Criterion Model 42, MTS). The MN patch was fixed onto a stainless‐steel base plate, with an initial gap of 0.1 mm between the needle tips and the top plate. The compression speed was maintained at 1 mm min^−1^, and both the compression force and displacement were continuously recorded until the force exceeded 100 N. For the skin penetration test, the MNs were applied to the HS tissue established in a rabbit ear model for 5 min. After removal of the MN patches, the HS samples were immediately embedded in OCT compound, frozen, sectioned using a cryotome (Cryostar NX50, Thermo), and subjected to H&E staining. The stained sections were then examined under the bright field of a fluorescence microscope.

### 4.15 Establishment of HS Model

The rabbit ear HS model was established according to a previously reported method [38]. Briefly, after induction of general anesthesia and thorough disinfection, four circular full‐thickness wounds (10 mm in diameter) were created on the ventral side of each ear using a dermal biopsy punch, extending down to the bare cartilage. The epidermis, dermis, and perichondrium were carefully excised, taking particular care to avoid cartilage injury during perichondrium removal. The wounds were disinfected daily. Ten days post‐surgery, the scabs were gently removed to delay re‐epithelialization and promote tissue proliferation. By Day 21, the wounds had completely healed, and the HS model was successfully established.

### 4.16 In Vivo Studies on Anti‐Scarring Effects

After establishment of the HS model, the rabbits were randomly assigned to five groups (n = 3 biologically independent animals per group; 12 HS lesions per group were generated from these rabbits to provide sufficient tissue for different downstream analyses): (1) Control group, untreated rabbits; (2) PDANP MNs(+) group, rabbits treated with PDANP‐loaded MNs and exposed to NIR irradiation at 0.25 W cm^−2^, with the photothermal‐induced maximum temperature maintained between 42°C and 45°C; (3) PDANP/Bro MNs(+) group, rabbits treated with MNs containing both PDANP and Bro under the same photothermal conditions as group (2); (4) PDA‐NO/Bro MNs group, rabbits treated with MNs containing PDA‐NO and Bro without NIR irradiation; and (5) PDA‐NO/Bro MNs(+) group, rabbits treated with MNs containing PDA‐NO and Bro under the same photothermal conditions as group (2). Beginning on Day 22, treatments were administered once weekly for three consecutive sessions, while scar photographs and body weights were recorded throughout the study period. On Day 43, samples were collected for H&E staining, Masson's trichrome staining, Sirius Red staining, and Western blot analysis. In addition, immunofluorescence staining for FN, α‐SMA, and Collagen I was performed and visualized using a fluorescence microscope.

### 4.17 Quantitative Analysis of SEI, Relative Collagen Content, and Type I/III Collagen Ratio

For in vivo histological quantification, one HS sample from each rabbit was used for each analysis, and the data were analyzed at the animal level (*n* = 3 biologically independent animals per group).

In H&E‐stained sections, the SEI was used to assess the degree of HS elevation according to the following formula:

(2) $$SEI=\frac{H}{H_{0}}$$

where *H* represents the vertical distance from the highest point of the HS surface to the cartilage surface, and *H*_0_ denotes the thickness of normal skin, measured from the stratum corneum (SC) to the cartilage surface [39].

The relative collagen content was quantitatively analyzed by extracting blue‐channel regions from Masson's trichrome‐stained sections using ImageJ (version 1.53t, RRID:SCR_003070), and expressed as the ratio of the blue area to the total tissue area. The type I/III collagen ratio was determined from Sirius Red‐stained sections. Sirius Red is a strongly acidic dye that binds to basic groups in collagen molecules (e.g., amino groups of amino acids), producing characteristic birefringent colors under polarized light. Type I collagen appears bright red or yellow due to its thick, tightly packed fibers with strong birefringence, whereas type III collagen appears green owing to its thinner fibers and weaker birefringence. ImageJ was employed to separate the red and green channels, extract the corresponding regions for type I and type III collagen, and quantify their relative areas to calculate the type I/III collagen ratio. Consistent image analysis parameters (e.g., threshold values) were applied across all channels and samples to ensure reproducibility.

### 4.18 Statistics Analysis

All experiments were conducted at least three times. Data are presented as the mean ± standard deviation (SD). Statistical analyses were performed using GraphPad Prism (version 9.4.1, RRID:SCR_002798). Comparisons among multiple groups were evaluated using one‐way analysis of variance (ANOVA), followed by Tukey's HSD post hoc test for pairwise comparisons. For comparisons between two groups, a two‐tailed Student's t‐test was applied. Statistical significance was defined as p < 0.05. The symbols ^*^ *p* < 0.05, ^**^ *p* < 0.01, ^***^ *p* < 0.001, and ^****^ *p* < 0.0001 indicate increasing levels of significance, while “ns” denotes no significant difference.

### 4.19 Manuscript Preparation

AI‐assisted tools were employed only for language polishing and grammar refinement, and were not used to generate or modify images, analyze data, or formulate the key arguments or conclusions of this study. Schematic illustrations and chemical structures were prepared with BioRender.com (web‐based platform), Autodesk 3ds Max (version 2025.2), Adobe Photoshop (version 12.0), and ChemDraw (version 22.0.0). Autodesk 3ds Max was available under valid Autodesk educational access, whereas the other tools were covered by valid paid licenses/subscriptions. These tools were applied solely for schematic illustration preparation, 3D rendering, chemical structure drawing, and figure layout, and not for processing or modifying experimental images or raw data. Their use complied with the corresponding licensing terms.

## Author Contributions

**Junzhe Fu**: conceptualization, methodology, investigation, validation, data curation, visualization, Writing – original draft, Writing – review and editing, formal analysis. **Yixian Mu**: investigation. **Fan Jia**: conceptualization, methodology, writing – review and editing, investigation. **Jian Ji**: supervision, resources, project administration, funding acquisition, writing – review and editing. **Dingqi Tang**: writing – review and editing. **Qiao Jin**: project administration, resources, funding acquisition, supervision. **Youxiang Wang**: supervision, resources, project administration, writing – review and editing, funding acquisition. **Wanzhe Liu**: investigation, validation .

## Funding

This research was supported by the “Leading Goose” R&D Program of Zhejiang (2025C04012), the Foundation of Transvascular Implantation Devices Research Institute (TIDRI) (KY012026003, KY012024008), and the Fundamental Research Funds for the Central Universities (226‐2026‐00056).

## Conflicts of Interest

The authors declare no conflicts of interest.

## Supporting information




**Supporting File**: advs77376‐sup‐0001‐SuppMat.docx.

## Data Availability

The data that support the findings of this study are available from the corresponding author upon reasonable request.
