# Supplementary material for: A Photothermally Amplified Enzyme–Nitric Oxide Co‐Regulatory System Reprograms Pathological ECM–Fibroblast Crosstalk to Alleviate Hypertrophic Scarring
Source: Adv Sci (Weinh). 2026 Aug 24;13(63):e77376. doi: 10.1002/advs.77376 (PMC13502348; doi:10.1002/advs.77376)
Supplement: Supplementary file 1 [file ADVS-13-e77376-s001.docx]

Supporting Information

A Photothermally Amplified Enzyme–Nitric Oxide Co-Regulatory System Reprograms Pathological ECM–Fibroblast Crosstalk to Alleviate Hypertrophic Scarring

Junzhe Fu, Fan Jia, Yixian Mu, Wanzhe Liu, Dingqi Tang, Qiao Jin, Jian Ji*, and Youxiang Wang*

J. Fu, F. Jia, Y. Mu, W. Liu, D. Tang, Prof. Q. Jin, Prof. J. Ji, Prof. Y. Wang

MOE Key Laboratory of Macromolecular Synthesis and Functionalization, Department of Polymer Science and Engineering, Zhejiang University, Hangzhou 310058, P. R. China.

Prof. J. Ji

State Key Laboratory of Transvascular Implantation Devices, The Second Affiliated Hospital, Zhejiang University School of Medicine, Hangzhou 310009, P. R. China.

E-mail: [jijian@zju.edu.cn](mailto:jijian@zju.edu.cn) (Prof. J. Ji), [yx_wang@zju.edu.cn](mailto:yx_wang@zju.edu.cn) (Prof. Y. Wang)

Supplementary Figures:

**Figure S1.** Detailed synthetic routes of PDA-NO through a two-step surface functionalization process.

**Figure S2.** Representative TEM image of the nanoparticles. (A) PDANP. (B) PDA-T.

**Figure S3.** UV-vis absorption spectrum of SNAP, showing a characteristic band at 340 nm.

**Figure S4.** Total releasable NO content of PDA-NO after storage in methanol at -20 °C under light-protected conditions for 1, 3, and 7 days, expressed relative to Day 0.

**Figure S5.** Representative photographs taken at key stages of the rabbit ear scar model construction.

**Figure S6.** Infrared thermal images of rabbit ear HS tissues treated with PDANP MNs under NIR irradiation at different power densities (0.25, 0.35, and 0.50 W cm^-2^).

**Figure S7.** Photographs of HS lesions at different time points, following a single photothermal treatment on Day 22 and continuous observation until Day 34 with natural eschar detachment.

**Figure S8.** Representative H&E staining of HS from Control and high-intensity photothermal-treated groups on Day 34.

**Figure S9.** Western blot bands showing TGF-β1 and α-SMA expression in each group.

**Figure S10.** Western blot bands showing Collagen I expression in each group.

**Figure S11.** Full-array bright-field stereomicroscopy image of PDA-NO/Bro MNs.

**Figure S12.** UV-vis absorption spectrum of the Bro–FITC conjugate, with a calculated degree of labeling (DOL) of 0.91.

**Figure S13.** Time-dependent dissolution process of PDA-NO/Bro MNs in PBS.

**Figure S14.** Photographs of PDA-NO/Bro MNs after insertion into HS tissue for different durations.

**Figure S15.** Body weight change of animals during the experimental period, expressed relative to the initial body weight. Each line represents an individual rabbit.

**Figure S16.** Western blot bands showing p-YAP, YAP, and TGF-β1 expression in each group.

**Figure S17.** ^1^H NMR spectrum of SNAP in CDCl_3_.

**Figure S18.** Calibration curve of NO-derived nitrite determined by the Griess reaction at 540 nm.
